# Supplementary figures and images for: Development of a dynamic prediction model for unplanned ICU admission and mortality in hospitalized patients
Source: PLOS Digit Health. 2023 Jun 9;2(6):e0000116. doi: 10.1371/journal.pdig.0000116 (PMC10256150; doi:10.1371/journal.pdig.0000116)

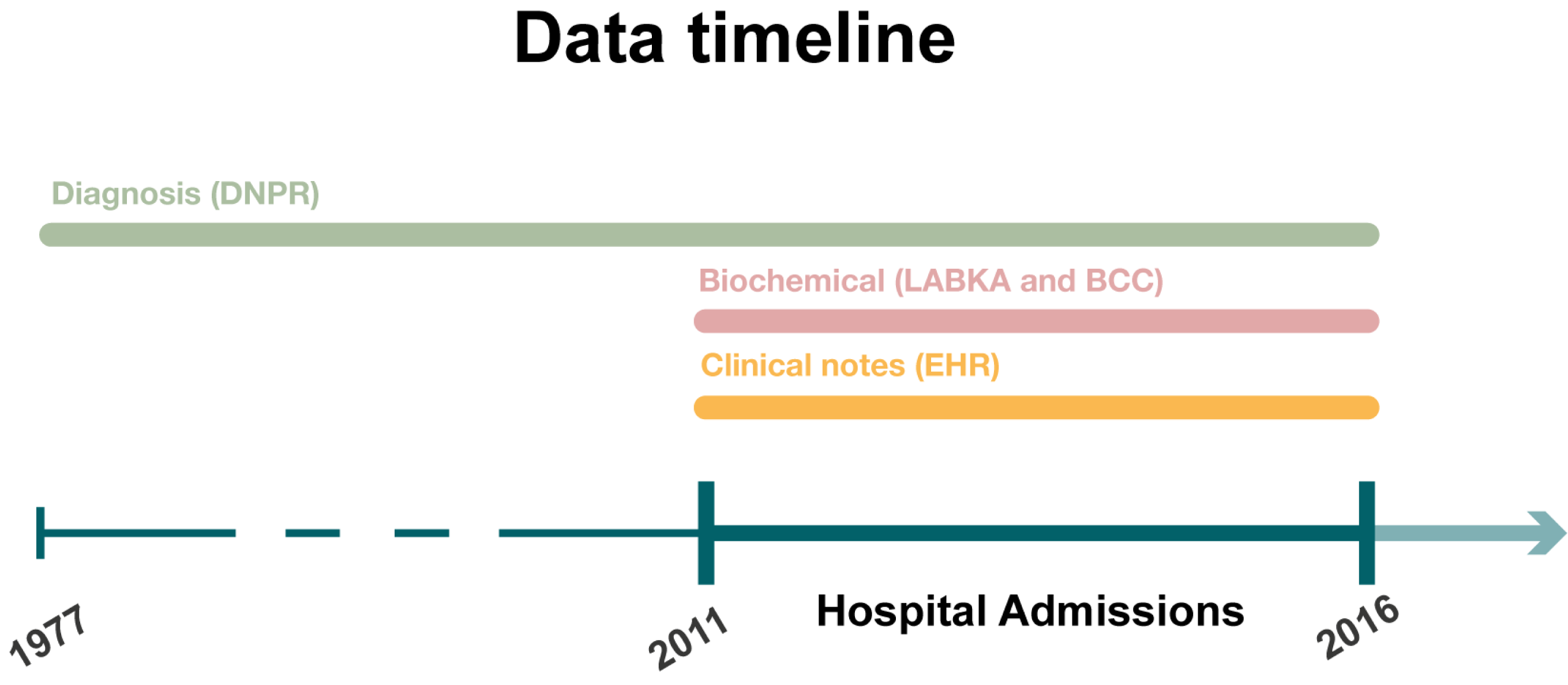

Supplement: S1 Fig — (TIF) [file pdig.0000116.s001.tif]

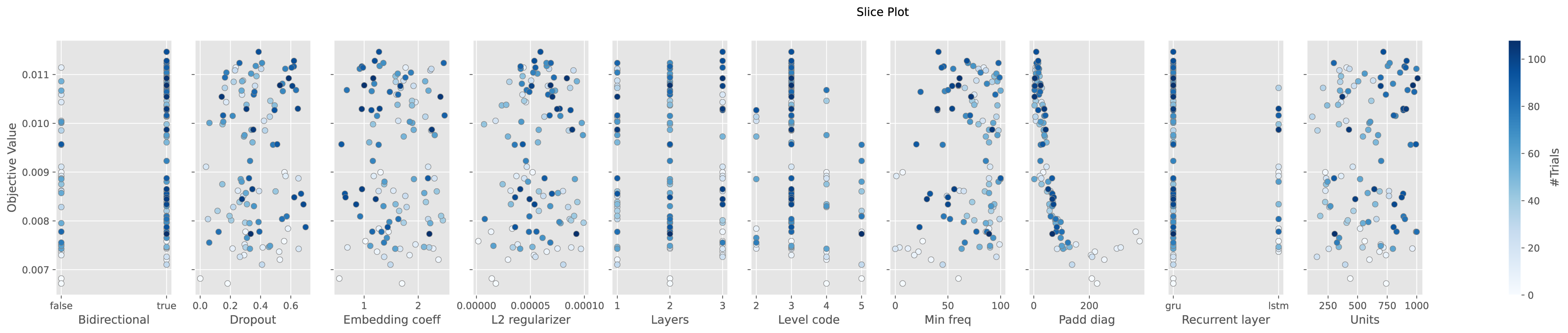

Supplement: S2 Fig — (TIF) [file pdig.0000116.s002.tif]

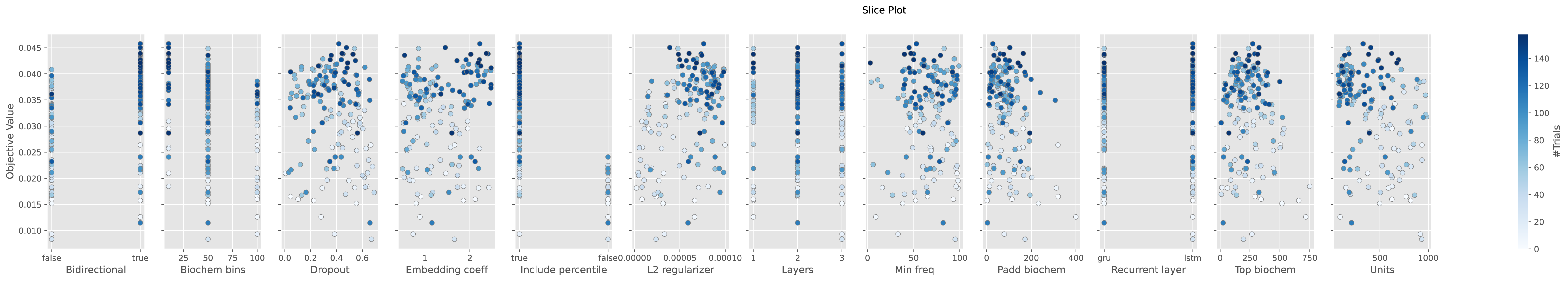

Supplement: S3 Fig — (TIF) [file pdig.0000116.s003.tif]

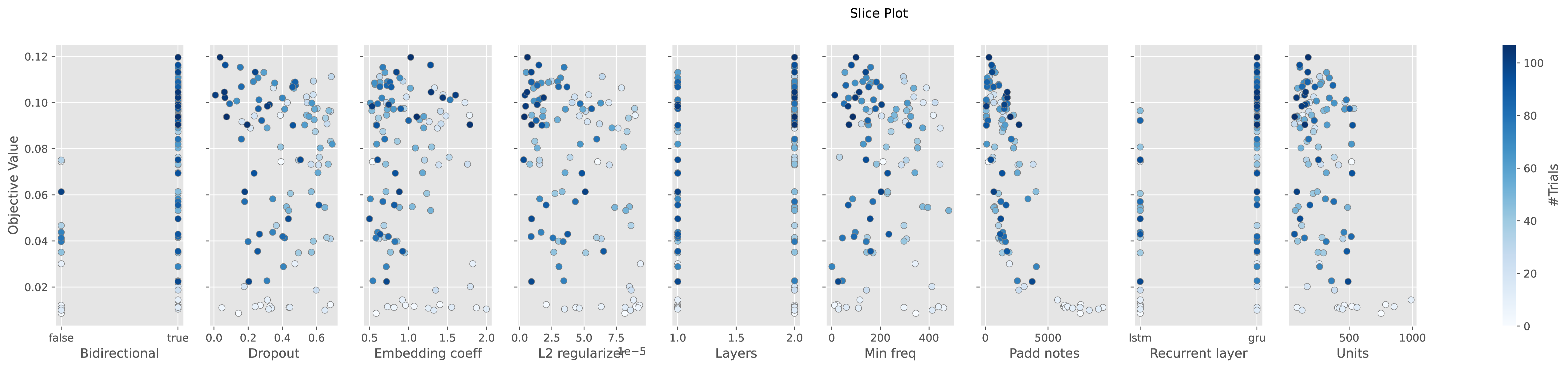

Supplement: S4 Fig — (TIF) [file pdig.0000116.s004.tif]

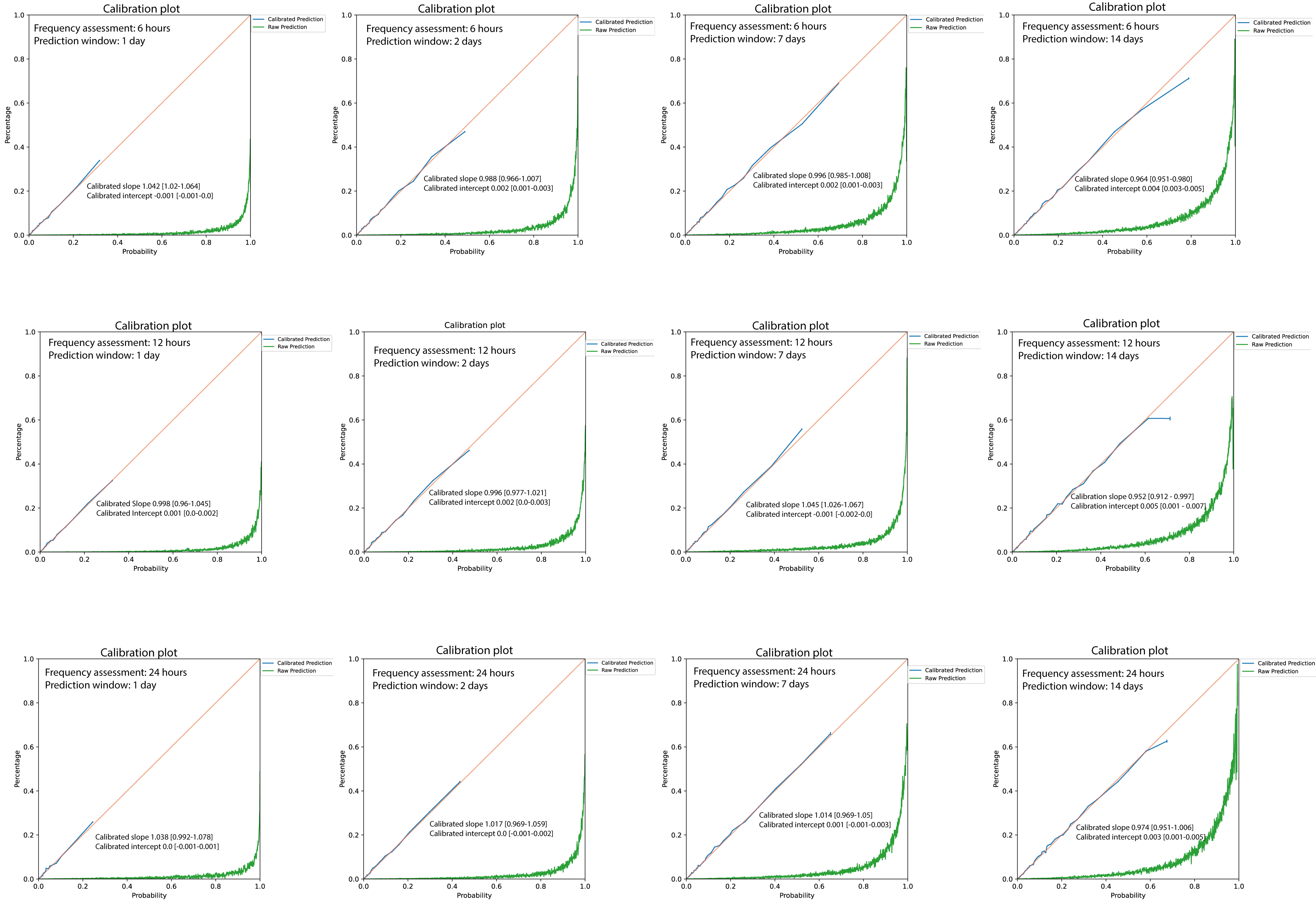

Supplement: S5 Fig — (TIF) [file pdig.0000116.s005.tif]

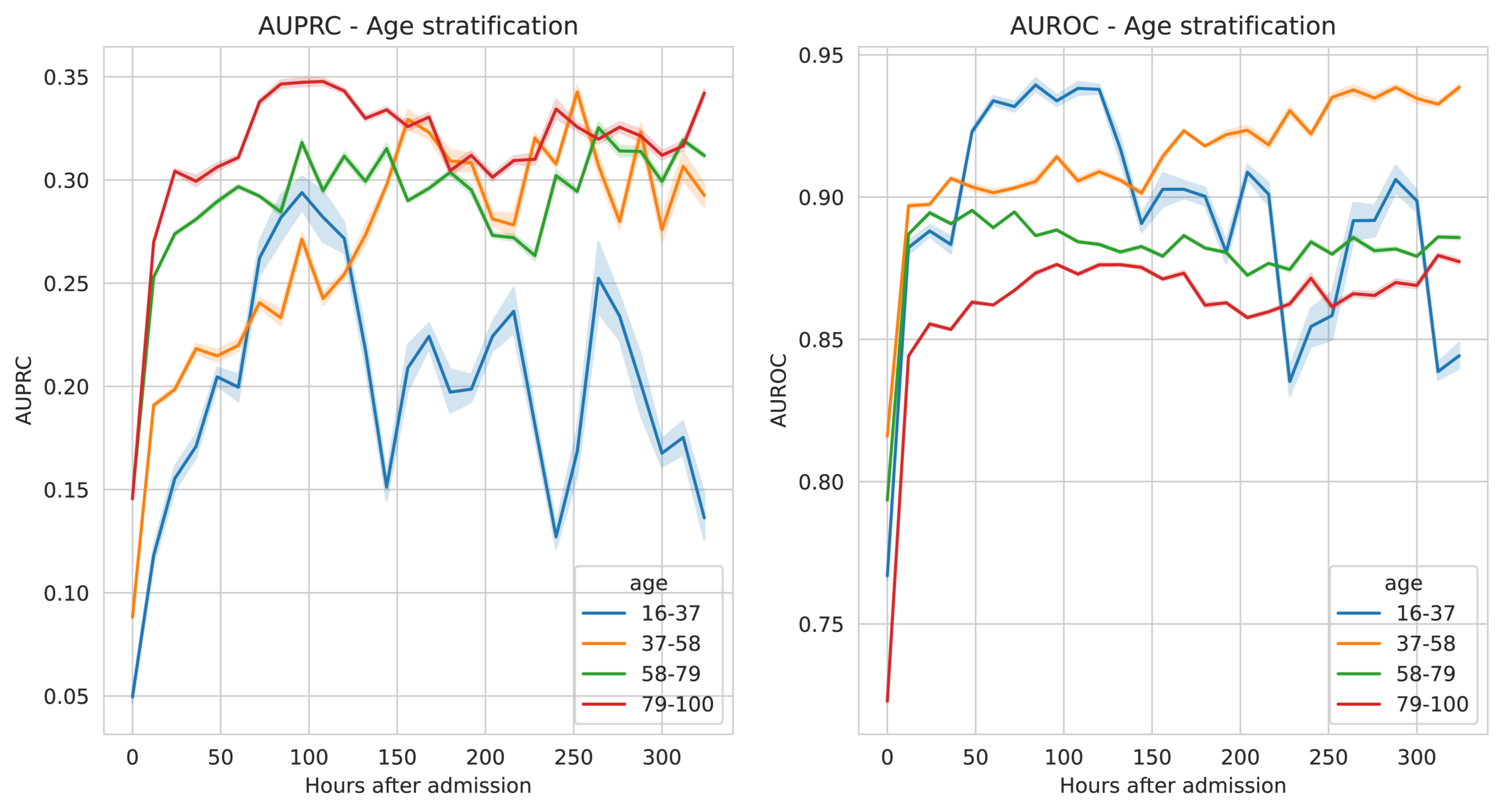

Supplement: S6 Fig — (TIF) [file pdig.0000116.s006.tif]

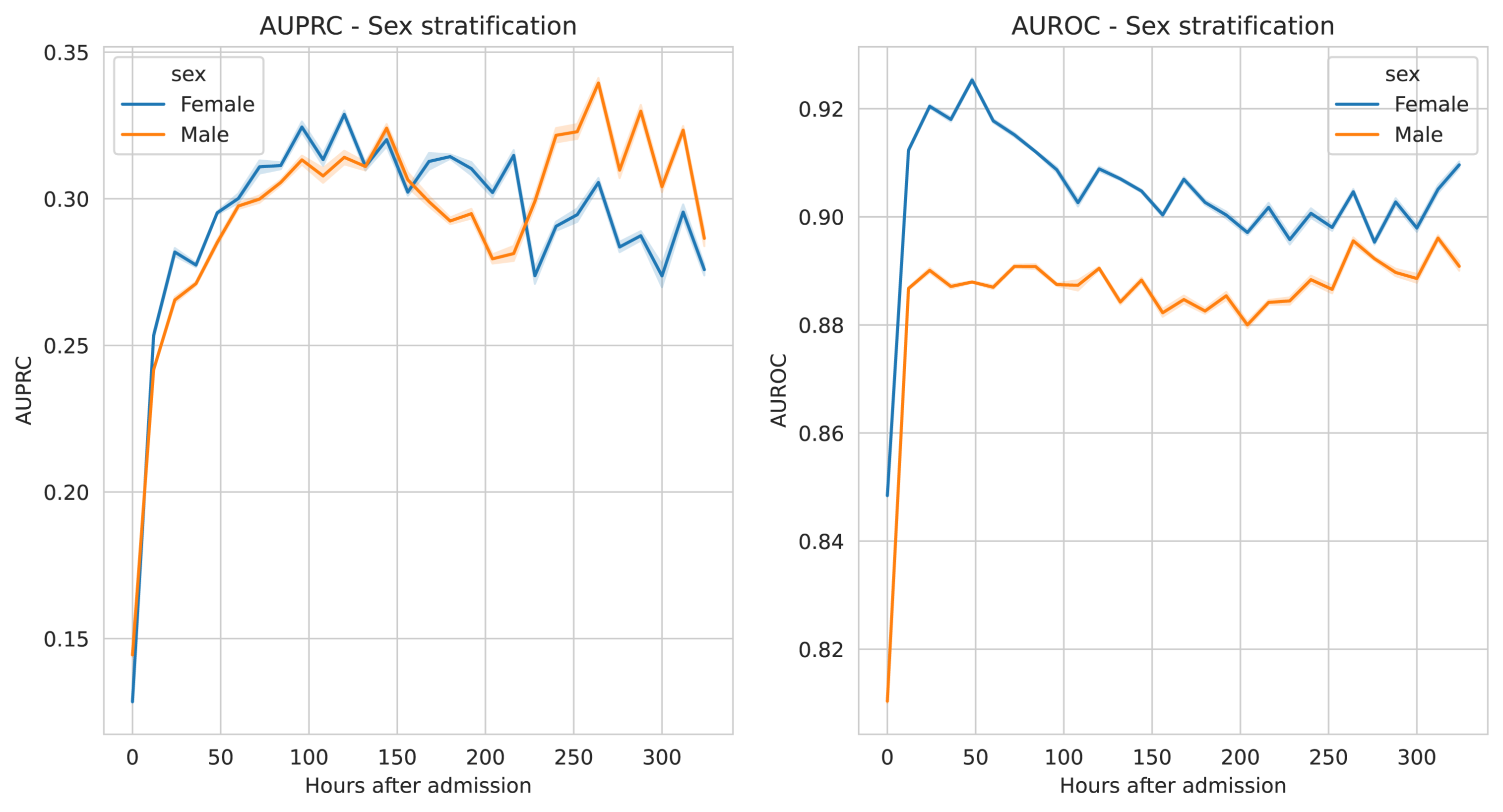

Supplement: S7 Fig — (TIF) [file pdig.0000116.s007.tif]

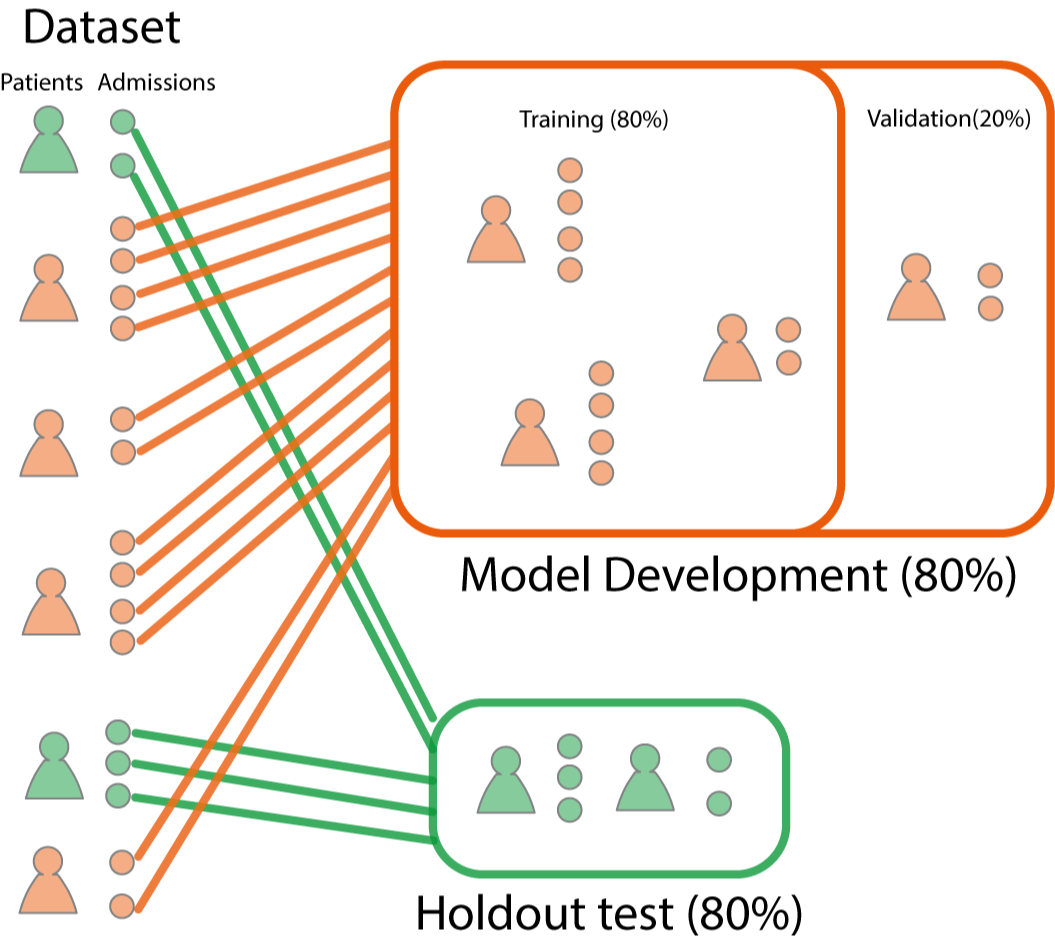

Supplement: S8 Fig — Patients are first assigned randomly to either development set or holdout test set (80 and 20%). The development set is used for hyperparameter search (80% assigned for training and 20% for internal validation); the holdout test is used for the assessment of models’ performances. (TIF) [file pdig.0000116.s008.tif]
