# Supplementary material for: Development of a dynamic prediction model for unplanned ICU admission and mortality in hospitalized patients
Source: PLOS Digit Health. 2023 Jun 9;2(6):e0000116. doi: 10.1371/journal.pdig.0000116 (PMC10256150; doi:10.1371/journal.pdig.0000116)
Supplement: S1 Table — We tested different combinations of the following parameters: bidirectional layer, units, layer, padding size for each data source, dropout, regularization, ICD level characters cut-off, biochemical percentile inclusion, top number of biochemical included, biochemical value. (PDF) [file pdig.0000116.s009.pdf]

TableS1: Hyperparameter selection

We tested different combinations of the following parameters: bidirectional layer, units, layer, padding size for each data source, dropout, regularization, ICD level characters cut-off, biochemical percentile inclusion, top number of biochemical included, biochemical value.

Model	Data sources	Parameters	Best Combination
Baseline Model	- Age at admission<br>- Sex<br>- Number of previous admissions	Random integer:<br>- Units [1-128]	- Unit 66
Diagnose Model	- Age at admission<br>- Sex<br>- Number of previous admissions<br>- Diagnoses	Random integer:<br>- Units [integer 64-1024]<br>- Padding Diagnoses [2,400]<br>- Min frequency [0,100]<br>Uniform Distribution:<br>- Dropout [0, 0.7]<br>- L2 regularizer [0,1e-4]<br>- Embedding coefficient [0.5-2.5]<br>Suggest Categorical:<br>- Bidirectional [False - True]<br>- Recurrent Layer [GRU-LSTM]<br>- Layers [1-2-3]<br>- Level code [2,3,4,5]	Random integer:<br>- Units 718<br>- Padding Diagnoses 9<br>- Min frequency 41<br>Uniform Distribution:<br>- Dropout 0.39<br>- L2 regularizer 6e-05<br>- Embedding coefficient 2.22<br>Suggest Categorical:<br>- Bidirectional True<br>- Recurrent Layer GRU<br>- Layers 3<br>- Level code 3
Biochemical Model	- Age at admission<br>- Sex<br>- Number of previous admissions<br>- Biochemical measurements	Random integer:<br>- Units [integer 64-1024]<br>- Padding Biochemicals [2,400]<br>- Top measurements [2,750]<br>- Min frequency [0,100]<br>Uniform Distribution:<br>- Dropout [0, 0.7]<br>- L2 regularizer [0,1e-4]<br>- Embedding coefficient [0.5-2.5]<br>Suggest Categorical:<br>- Bidirectional [False - True]<br>- Layers [1-2-3]	Random integer:<br>- Units 325<br>- Padding Biochemicals 28<br>- Top measurements 273<br>- Min frequency 53<br>Uniform Distribution:<br>- Dropout 0.41<br>- L2 regularizer 7.6e-05<br>- Embedding coefficient 2.22<br>Suggest Categorical:<br>- Bidirectional True<br>- Layers 3<br>- Include measurement value True

		- Include measurement value [False - True]<br>- Measurement value percentile [10,50,100]<br>- Recurrent Layer [GRU-LSTM]	- Measurement value percentile 10<br>- Recurrent Layer LSTM
Notes Model	- Age at admission<br>- Sex<br>- Number of previous admissions<br>- Medical Notes	Random integer:<br>- Units [integer 64-1024]<br>- Padding notes [50, 10000]<br>- Min frequency [0,500]<br>Uniform Distribution:<br>- Dropout [0, 0.7]<br>- L2 regularizer [0,1e-4]<br>- Embedding coefficient [0.5-2]<br>Suggest Categorical:<br>- Bidirectional [False - True]<br>- Layers [1-2]<br>- Recurrent Layer [GRU-LSTM]	Random integer:<br>- Units 177<br>- Padding notes 299<br>- Min frequency 100<br>Uniform Distribution:<br>- Dropout 0.03<br>- L2 regularizer 6.02e-06<br>- Embedding coefficient 1.02<br>Suggest Categorical:<br>- Bidirectional True<br>- Layers 2<br>- Recurrent Layer GRU
EHR Model	- Age at admission<br>- Sex<br>- Number of previous admissions<br>- Diagnoses<br>- Biochemical measurements<br>- Medical Notes	Prediction window (H): [24,48,168,336]<br>Assessment rate (H): [6,12,24]	Prediction window (H): 336<br>Assessment rate (H): 6
