# Supplementary material for: Development of a dynamic prediction model for unplanned ICU admission and mortality in hospitalized patients
Source: PLOS Digit Health. 2023 Jun 9;2(6):e0000116. doi: 10.1371/journal.pdig.0000116 (PMC10256150; doi:10.1371/journal.pdig.0000116)
Supplement: S2 Table — The metrics are selected for different risk thresholds [1%, 5%, 10%, 20% and 50%]. Statistics are provided as median and 95% confidence interval: (median, 2.5% percentile, 97.5% percentile). (PDF) [file pdig.0000116.s010.pdf]

Supplementary metrics

Prediction window	Frequency of assessment	Risk	Precision/PPV	Recall/TPR	Specificity
1d	6h	0.01	(0.042, 0.041, 0.043)	(0.739, 0.735, 0.744)	(0.93, 0.93, 0.93)
		0.05	(0.115, 0.113, 0.117)	(0.471, 0.466, 0.476)	(0.985, 0.985, 0.985)
		0.1	(0.15, 0.147, 0.152)	(0.375, 0.37, 0.379)	(0.991, 0.991, 0.991)
		0.2	(0.198, 0.194, 0.202)	(0.27, 0.265, 0.274)	(0.995, 0.995, 0.996)
		0.5	(0.337, 0.327, 0.349)	(0.06, 0.058, 0.063)	(0.999, 0.999, 0.999)
	12h	0.01	(0.041, 0.04, 0.042)	(0.709, 0.7, 0.715)	(0.93, 0.93, 0.931)
		0.05	(0.107, 0.105, 0.109)	(0.45, 0.442, 0.457)	(0.984, 0.984, 0.984)
		0.1	(0.153, 0.149, 0.156)	(0.32, 0.312, 0.326)	(0.993, 0.992, 0.993)
		0.2	(0.198, 0.193, 0.204)	(0.215, 0.209, 0.221)	(0.996, 0.996, 0.996)
		0.5	(0.323, 0.304, 0.347)	(0.036, 0.034, 0.04)	(0.999, 0.999, 0.999)
	1d	0.01	(0.039, 0.039, 0.04)	(0.666, 0.657, 0.674)	(0.93, 0.93, 0.93)
		0.05	(0.103, 0.1, 0.105)	(0.406, 0.397, 0.416)	(0.985, 0.985, 0.985)
		0.1	(0.134, 0.129, 0.139)	(0.313, 0.305, 0.322)	(0.991, 0.991, 0.991)
		0.2	(0.185, 0.178, 0.193)	(0.203, 0.194, 0.211)	(0.996, 0.996, 0.996)
		0.5	(0.259, 0.244, 0.273)	(0.09, 0.084, 0.096)	(0.999, 0.999, 0.999)
2d	6h	0.01	(0.048, 0.048, 0.048)	(0.812, 0.809, 0.816)	(0.878, 0.878, 0.878)
		0.05	(0.13, 0.128, 0.131)	(0.54, 0.536, 0.544)	(0.972, 0.972, 0.973)
		0.1	(0.184, 0.182, 0.186)	(0.405, 0.402, 0.41)	(0.986, 0.986, 0.986)
		0.2	(0.27, 0.266, 0.273)	(0.246, 0.243, 0.25)	(0.995, 0.995, 0.995)
		0.5	(0.468, 0.45, 0.488)	(0.019, 0.017, 0.02)	(0.999, 0.999, 0.999)
	12h	0.01	(0.042, 0.041, 0.042)	(0.817, 0.812, 0.82)	(0.858, 0.857, 0.858)
		0.05	(0.125, 0.123, 0.127)	(0.542, 0.536, 0.547)	(0.971, 0.971, 0.971)
		0.1	(0.191, 0.188, 0.195)	(0.387, 0.382, 0.393)	(0.988, 0.987, 0.988)
		0.2	(0.259, 0.254, 0.264)	(0.274, 0.268, 0.279)	(0.994, 0.994, 0.994)
		0.5	(0.462, 0.44, 0.479)	(0.038, 0.036, 0.039)	(0.999, 0.999, 0.999)
	1d	0.01	(0.04, 0.039, 0.041)	(0.779, 0.772, 0.785)	(0.857, 0.857, 0.858)
		0.05	(0.121, 0.118, 0.123)	(0.496, 0.489, 0.503)	(0.972, 0.972, 0.973)
		0.1	(0.187, 0.182, 0.192)	(0.348, 0.34, 0.355)	(0.988, 0.988, 0.989)
		0.2	(0.234, 0.228, 0.24)	(0.264, 0.258, 0.271)	(0.993, 0.993, 0.993)
		0.5	(0.443, 0.413, 0.474)	(0.026, 0.024, 0.029)	(0.999, 0.999, 0.999)
1w	6h	0.01	(0.061, 0.061, 0.062)	(0.913, 0.912, 0.915)	(0.729, 0.729, 0.729)
		0.05	(0.14, 0.14, 0.141)	(0.706, 0.704, 0.708)	(0.916, 0.916, 0.916)
		0.1	(0.217, 0.216, 0.219)	(0.537, 0.534, 0.54)	(0.962, 0.962, 0.963)
		0.2	(0.316, 0.314, 0.318)	(0.362, 0.359, 0.364)	(0.985, 0.985, 0.985)
		0.5	(0.455, 0.451, 0.459)	(0.164, 0.162, 0.165)	(0.996, 0.996, 0.996)
	12h	0.01	(0.055, 0.055, 0.056)	(0.899, 0.897, 0.901)	(0.704, 0.703, 0.704)
		0.05	(0.138, 0.137, 0.139)	(0.66, 0.656, 0.663)	(0.921, 0.92, 0.921)
		0.1	(0.196, 0.195, 0.198)	(0.52, 0.517, 0.523)	(0.959, 0.959, 0.959)
		0.2	(0.263, 0.261, 0.266)	(0.366, 0.362, 0.37)	(0.98, 0.98, 0.98)
		0.5	(0.407, 0.401, 0.413)	(0.139, 0.136, 0.141)	(0.996, 0.996, 0.996)
	1d	0.01	(0.052, 0.052, 0.053)	(0.902, 0.9, 0.905)	(0.69, 0.69, 0.691)
		0.05	(0.14, 0.139, 0.142)	(0.626, 0.621, 0.63)	(0.928, 0.927, 0.928)
		0.1	(0.208, 0.205, 0.21)	(0.479, 0.474, 0.483)	(0.966, 0.965, 0.966)
		0.2	(0.3, 0.296, 0.304)	(0.304, 0.3, 0.309)	(0.987, 0.986, 0.987)
		0.5	(0.456, 0.446, 0.464)	(0.112, 0.109, 0.115)	(0.997, 0.997, 0.998)
		0.01	(0.067, 0.067, 0.067)	(0.942, 0.941, 0.943)	(0.632, 0.632, 0.633)

2w	6h	0.05	(0.147, 0.146, 0.148)	(0.756, 0.754, 0.758)	(0.877, 0.877, 0.878)
		0.1	(0.214, 0.213, 0.215)	(0.602, 0.6, 0.604)	(0.938, 0.938, 0.938)
		0.2	(0.319, 0.318, 0.321)	(0.381, 0.379, 0.383)	(0.977, 0.977, 0.977)
		0.5	(0.512, 0.507, 0.517)	(0.105, 0.103, 0.106)	(0.997, 0.997, 0.997)
	12h	0.01	(0.063, 0.062, 0.063)	(0.946, 0.945, 0.947)	(0.609, 0.608, 0.609)
		0.05	(0.146, 0.145, 0.147)	(0.744, 0.741, 0.747)	(0.88, 0.88, 0.88)
		0.1	(0.219, 0.217, 0.22)	(0.58, 0.578, 0.583)	(0.943, 0.942, 0.943)
		0.2	(0.31, 0.307, 0.312)	(0.397, 0.395, 0.4)	(0.976, 0.975, 0.976)
		0.5	(0.535, 0.524, 0.542)	(0.084, 0.083, 0.086)	(0.998, 0.998, 0.998)
	1d	0.01	(0.061, 0.06, 0.061)	(0.938, 0.936, 0.94)	(0.61, 0.61, 0.611)
		0.05	(0.143, 0.142, 0.144)	(0.705, 0.702, 0.709)	(0.886, 0.885, 0.886)
		0.1	(0.21, 0.208, 0.212)	(0.546, 0.542, 0.55)	(0.945, 0.944, 0.945)
		0.2	(0.298, 0.295, 0.302)	(0.344, 0.341, 0.348)	(0.978, 0.978, 0.978)
		0.5	(0.487, 0.476, 0.5)	(0.071, 0.069, 0.073)	(0.998, 0.998, 0.998)
