# Supplementary material for: Development of a dynamic prediction model for unplanned ICU admission and mortality in hospitalized patients
Source: PLOS Digit Health. 2023 Jun 9;2(6):e0000116. doi: 10.1371/journal.pdig.0000116 (PMC10256150; doi:10.1371/journal.pdig.0000116)
Supplement: S3 Table — Given the same prediction window, the statistics for the models at lower frequency of assessment (12 and 24 hours) would be the same as the model presented in this table (e.g., the model with a frequency of assessment of 12 hours and a prediction window of 14 days has the same statistics of the model having the same prediction window and a frequency of assessment of 6 hours). (PDF) [file pdig.0000116.s011.pdf]

Count of patients/admissions/positive cases for 12 models using all data

Frequency of assessment	Prediction window	Time of assessment	Data split	Number of Patients	Number of admissions	Number of positive cases
**6**	**24**	0	train	682041	1773759	9319
**6**	**24**	0	test	170579	445620	2432
**6**	**24**	6	train	595230	1430806	6336
**6**	**24**	6	test	148815	359301	1682
**6**	**24**	12	train	536198	1244785	5324
**6**	**24**	12	test	133857	312142	1408
**6**	**24**	18	train	506713	1147199	4708
**6**	**24**	18	test	126598	287821	1175
**6**	**24**	24	train	470286	1026484	4205
**6**	**24**	24	test	117514	257349	1037
**6**	**24**	30	train	427620	900052	3839
**6**	**24**	30	test	107150	225732	922
**6**	**24**	36	train	406952	846068	3477
**6**	**24**	36	test	102015	212119	834
**6**	**24**	42	train	393103	806893	3181
**6**	**24**	42	test	98503	202406	783
**6**	**24**	48	train	373388	749902	3023
**6**	**24**	48	test	93504	188225	740
**6**	**24**	54	train	343796	677617	2844
**6**	**24**	54	test	86068	170102	713
**6**	**24**	60	train	327267	644880	2754
**6**	**24**	60	test	81954	161872	686
**6**	**24**	66	train	315030	617864	2630
**6**	**24**	66	test	78805	155133	682
**6**	**24**	72	train	298362	577472	2524
**6**	**24**	72	test	74603	145004	615
**6**	**24**	78	train	277859	532045	2450
**6**	**24**	78	test	69373	133613	587
**6**	**24**	84	train	266872	510652	2259
**6**	**24**	84	test	66602	128123	566
**6**	**24**	90	train	258273	491220	2164
**6**	**24**	90	test	64465	123099	531
**6**	**24**	96	train	245476	460221	2007
**6**	**24**	96	test	61266	115360	538
**6**	**24**	102	train	230507	427069	1915
**6**	**24**	102	test	57570	107056	552
**6**	**24**	108	train	224304	414025	1820
**6**	**24**	108	test	56055	103809	516
**6**	**24**	114	train	218128	399504	1767
**6**	**24**	114	test	54488	100198	481
**6**	**24**	120	train	208579	376810	1734
**6**	**24**	120	test	52084	94556	482
**6**	**24**	126	train	198380	354896	1663
**6**	**24**	126	test	49598	89216	441
**6**	**24**	132	train	194281	346231	1649
**6**	**24**	132	test	48595	87068	444
**6**	**24**	138	train	189601	335212	1544
**6**	**24**	138	test	47450	84279	430
**6**	**24**	144	train	181891	317037	1465
**6**	**24**	144	test	45542	79730	400
**6**	**24**	150	train	174289	300273	1453
**6**	**24**	150	test	43675	75626	390
**6**	**24**	156	train	171297	294002	1381
**6**	**24**	156	test	42961	74075	355
**6**	**24**	162	train	167556	285303	1365
**6**	**24**	162	test	42022	71882	347
**6**	**24**	168	train	160558	269256	1314
**6**	**24**	168	test	40263	67796	324
**6**	**24**	174	train	153786	254423	1246
**6**	**24**	174	test	38568	64092	304
**6**	**24**	180	train	151332	249432	1224
**6**	**24**	180	test	37972	62757	297
**6**	**24**	186	train	148059	242325	1186
**6**	**24**	186	test	37211	61047	310
**6**	**24**	192	train	142529	230157	1166
**6**	**24**	192	test	35840	57986	298
**6**	**24**	198	train	137562	219792	1099
**6**	**24**	198	test	34656	55452	271
**6**	**24**	204	train	135716	216045	1093
**6**	**24**	204	test	34140	54453	247
**6**	**24**	210	train	132996	210342	1052
**6**	**24**	210	test	33450	53001	223
**6**	**24**	216	train	128616	201107	1008

**6**	**24**	216	test	32413	50774	211
**6**	**24**	222	train	124902	193653	984
**6**	**24**	222	test	31469	48941	213
**6**	**24**	228	train	123366	190727	958
**6**	**24**	228	test	31078	48215	205
**6**	**24**	234	train	121165	186131	926
**6**	**24**	234	test	30529	47120	201
**6**	**24**	240	train	117312	178265	866
**6**	**24**	240	test	29580	45191	195
**6**	**24**	246	train	114069	171923	856
**6**	**24**	246	test	28760	43594	213
**6**	**24**	252	train	112721	169428	846
**6**	**24**	252	test	28460	42989	210
**6**	**24**	258	train	110669	165490	767
**6**	**24**	258	test	27970	41993	210
**6**	**24**	264	train	107363	158988	743
**6**	**24**	264	test	27122	40329	207
**6**	**24**	270	train	104592	153895	728
**6**	**24**	270	test	26399	38991	184
**6**	**24**	276	train	103458	151874	719
**6**	**24**	276	test	26093	38475	193
**6**	**24**	282	train	101827	148752	737
**6**	**24**	282	test	25644	37637	168
**6**	**24**	288	train	98978	143428	735
**6**	**24**	288	test	24930	36297	168
**6**	**24**	294	train	96678	139415	706
**6**	**24**	294	test	24359	35325	169
**6**	**24**	300	train	95693	137726	654
**6**	**24**	300	test	24153	34926	166
**6**	**24**	306	train	94117	134889	624
**6**	**24**	306	test	23791	34251	165
**6**	**24**	312	train	91422	129977	603
**6**	**24**	312	test	23077	33009	154
**6**	**24**	318	train	89075	126050	581
**6**	**24**	318	test	22509	32055	144
**6**	**24**	324	train	88191	124601	581
**6**	**24**	324	test	22280	31651	145
**6**	**24**	330	train	86611	122009	601
**6**	**24**	330	test	21911	30987	146
**6**	**24**	336	train	83774	117018	564
**6**	**24**	336	test	21198	29746	144
**6**	**24**	342	train	81567	113112	544
**6**	**24**	342	test	20616	28723	127
**6**	**24**	348	train	80726	111734	525
**6**	**24**	348	test	20412	28378	130
**6**	**24**	354	train	79350	109408	492
**6**	**24**	354	test	20090	27822	129
**6**	**48**	0	train	682041	1773759	13674
**6**	**48**	0	test	170579	445620	3495
**6**	**48**	6	train	595230	1430806	10277
**6**	**48**	6	test	148815	359301	2632
**6**	**48**	12	train	536198	1244785	8904
**6**	**48**	12	test	133857	312142	2268
**6**	**48**	18	train	506713	1147199	7993
**6**	**48**	18	test	126598	287821	1988
**6**	**48**	24	train	470286	1026484	7315
**6**	**48**	24	test	117514	257349	1805
**6**	**48**	30	train	427620	900052	6764
**6**	**48**	30	test	107150	225732	1651
**6**	**48**	36	train	406952	846068	6309
**6**	**48**	36	test	102015	212119	1545
**6**	**48**	42	train	393103	806893	5879
**6**	**48**	42	test	98503	202406	1490
**6**	**48**	48	train	373388	749902	5612
**6**	**48**	48	test	93504	188225	1374
**6**	**48**	54	train	343796	677617	5386
**6**	**48**	54	test	86068	170102	1324
**6**	**48**	60	train	327267	644880	5082
**6**	**48**	60	test	81954	161872	1265
**6**	**48**	66	train	315030	617864	4865
**6**	**48**	66	test	78805	155133	1233
**6**	**48**	72	train	298362	577472	4612
**6**	**48**	72	test	74603	145004	1169
**6**	**48**	78	train	277859	532045	4416
**6**	**48**	78	test	69373	133613	1154

6	48	84	train	266872	510652	4137
6	48	84	test	66602	128123	1100
6	48	90	train	258273	491220	4001
6	48	90	test	64465	123099	1035
6	48	96	train	245476	460221	3794
6	48	96	test	61266	115360	1038
6	48	102	train	230507	427069	3625
6	48	102	test	57570	107056	1006
6	48	108	train	224304	414025	3520
6	48	108	test	56055	103809	976
6	48	114	train	218128	399504	3372
6	48	114	test	54488	100198	925
6	48	120	train	208579	376810	3237
6	48	120	test	52084	94556	896
6	48	126	train	198380	354896	3164
6	48	126	test	49598	89216	849
6	48	132	train	194281	346231	3078
6	48	132	test	48595	87068	816
6	48	138	train	189601	335212	2947
6	48	138	test	47450	84279	788
6	48	144	train	181891	317037	2840
6	48	144	test	45542	79730	744
6	48	150	train	174289	300273	2742
6	48	150	test	43675	75626	704
6	48	156	train	171297	294002	2637
6	48	156	test	42961	74075	665
6	48	162	train	167556	285303	2595
6	48	162	test	42022	71882	665
6	48	168	train	160558	269256	2528
6	48	168	test	40263	67796	626
6	48	174	train	153786	254423	2394
6	48	174	test	38568	64092	583
6	48	180	train	151332	249432	2357
6	48	180	test	37972	62757	555
6	48	186	train	148059	242325	2281
6	48	186	test	37211	61047	539
6	48	192	train	142529	230157	2222
6	48	192	test	35840	57986	513
6	48	198	train	137562	219792	2118
6	48	198	test	34656	55452	493
6	48	204	train	135716	216045	2078
6	48	204	test	34140	54453	461
6	48	210	train	132996	210342	2017
6	48	210	test	33450	53001	433
6	48	216	train	128616	201107	1912
6	48	216	test	32413	50774	419
6	48	222	train	124902	193653	1880
6	48	222	test	31469	48941	428
6	48	228	train	123366	190727	1830
6	48	228	test	31078	48215	422
6	48	234	train	121165	186131	1722
6	48	234	test	30529	47120	421
6	48	240	train	117312	178265	1634
6	48	240	test	29580	45191	410
6	48	246	train	114069	171923	1609
6	48	246	test	28760	43594	409
6	48	252	train	112721	169428	1588
6	48	252	test	28460	42989	408
6	48	258	train	110669	165490	1533
6	48	258	test	27970	41993	387
6	48	264	train	107363	158988	1503
6	48	264	test	27122	40329	380
6	48	270	train	104592	153895	1461
6	48	270	test	26399	38991	363
6	48	276	train	103458	151874	1393
6	48	276	test	26093	38475	361
6	48	282	train	101827	148752	1380
6	48	282	test	25644	37637	339
6	48	288	train	98978	143428	1372
6	48	288	test	24930	36297	334
6	48	294	train	96678	139415	1315
6	48	294	test	24359	35325	322
6	48	300	train	95693	137726	1260
6	48	300	test	24153	34926	312
6	48	306	train	94117	134889	1246

**6**	**48**	306	test	23791	34251	314
**6**	**48**	312	train	91422	129977	1190
**6**	**48**	312	test	23077	33009	305
**6**	**48**	318	train	89075	126050	1152
**6**	**48**	318	test	22509	32055	280
**6**	**48**	324	train	88191	124601	1128
**6**	**48**	324	test	22280	31651	279
**6**	**48**	330	train	86611	122009	1117
**6**	**48**	330	test	21911	30987	277
**6**	**48**	336	train	83774	117018	1081
**6**	**48**	336	test	21198	29746	258
**6**	**48**	342	train	81567	113112	1051
**6**	**48**	342	test	20616	28723	245
**6**	**48**	348	train	80726	111734	1035
**6**	**48**	348	test	20412	28378	245
**6**	**48**	354	train	79350	109408	996
**6**	**48**	354	test	20090	27822	232
**6**	**168**	0	train	682041	1773759	24751
**6**	**168**	0	test	170579	445620	6365
**6**	**168**	6	train	595230	1430806	20921
**6**	**168**	6	test	148815	359301	5401
**6**	**168**	12	train	536198	1244785	19071
**6**	**168**	12	test	133857	312142	4924
**6**	**168**	18	train	506713	1147199	17771
**6**	**168**	18	test	126598	287821	4552
**6**	**168**	24	train	470286	1026484	16657
**6**	**168**	24	test	117514	257349	4251
**6**	**168**	30	train	427620	900052	15772
**6**	**168**	30	test	107150	225732	4005
**6**	**168**	36	train	406952	846068	14900
**6**	**168**	36	test	102015	212119	3800
**6**	**168**	42	train	393103	806893	14189
**6**	**168**	42	test	98503	202406	3665
**6**	**168**	48	train	373388	749902	13579
**6**	**168**	48	test	93504	188225	3488
**6**	**168**	54	train	343796	677617	13000
**6**	**168**	54	test	86068	170102	3346
**6**	**168**	60	train	327267	644880	12478
**6**	**168**	60	test	81954	161872	3199
**6**	**168**	66	train	315030	617864	12035
**6**	**168**	66	test	78805	155133	3086
**6**	**168**	72	train	298362	577472	11547
**6**	**168**	72	test	74603	145004	2944
**6**	**168**	78	train	277859	532045	11083
**6**	**168**	78	test	69373	133613	2831
**6**	**168**	84	train	266872	510652	10640
**6**	**168**	84	test	66602	128123	2714
**6**	**168**	90	train	258273	491220	10299
**6**	**168**	90	test	64465	123099	2594
**6**	**168**	96	train	245476	460221	9846
**6**	**168**	96	test	61266	115360	2521
**6**	**168**	102	train	230507	427069	9478
**6**	**168**	102	test	57570	107056	2444
**6**	**168**	108	train	224304	414025	9195
**6**	**168**	108	test	56055	103809	2347
**6**	**168**	114	train	218128	399504	8861
**6**	**168**	114	test	54488	100198	2260
**6**	**168**	120	train	208579	376810	8554
**6**	**168**	120	test	52084	94556	2180
**6**	**168**	126	train	198380	354896	8269
**6**	**168**	126	test	49598	89216	2075
**6**	**168**	132	train	194281	346231	8066
**6**	**168**	132	test	48595	87068	2013
**6**	**168**	138	train	189601	335212	7799
**6**	**168**	138	test	47450	84279	1942
**6**	**168**	144	train	181891	317037	7542
**6**	**168**	144	test	45542	79730	1857
**6**	**168**	150	train	174289	300273	7291
**6**	**168**	150	test	43675	75626	1795
**6**	**168**	156	train	171297	294002	7043
**6**	**168**	156	test	42961	74075	1720
**6**	**168**	162	train	167556	285303	6860
**6**	**168**	162	test	42022	71882	1672
**6**	**168**	168	train	160558	269256	6653
**6**	**168**	168	test	40263	67796	1603

**6**	**168**	174	train	153786	254423	6404
**6**	**168**	174	test	38568	64092	1548
**6**	**168**	180	train	151332	249432	6236
**6**	**168**	180	test	37972	62757	1498
**6**	**168**	186	train	148059	242325	6073
**6**	**168**	186	test	37211	61047	1466
**6**	**168**	192	train	142529	230157	5878
**6**	**168**	192	test	35840	57986	1426
**6**	**168**	198	train	137562	219792	5680
**6**	**168**	198	test	34656	55452	1372
**6**	**168**	204	train	135716	216045	5519
**6**	**168**	204	test	34140	54453	1324
**6**	**168**	210	train	132996	210342	5360
**6**	**168**	210	test	33450	53001	1281
**6**	**168**	216	train	128616	201107	5181
**6**	**168**	216	test	32413	50774	1238
**6**	**168**	222	train	124902	193653	5053
**6**	**168**	222	test	31469	48941	1210
**6**	**168**	228	train	123366	190727	4909
**6**	**168**	228	test	31078	48215	1183
**6**	**168**	234	train	121165	186131	4773
**6**	**168**	234	test	30529	47120	1152
**6**	**168**	240	train	117312	178265	4603
**6**	**168**	240	test	29580	45191	1125
**6**	**168**	246	train	114069	171923	4491
**6**	**168**	246	test	28760	43594	1101
**6**	**168**	252	train	112721	169428	4361
**6**	**168**	252	test	28460	42989	1082
**6**	**168**	258	train	110669	165490	4239
**6**	**168**	258	test	27970	41993	1059
**6**	**168**	264	train	107363	158988	4128
**6**	**168**	264	test	27122	40329	1032
**6**	**168**	270	train	104592	153895	4017
**6**	**168**	270	test	26399	38991	992
**6**	**168**	276	train	103458	151874	3887
**6**	**168**	276	test	26093	38475	968
**6**	**168**	282	train	101827	148752	3810
**6**	**168**	282	test	25644	37637	938
**6**	**168**	288	train	98978	143428	3712
**6**	**168**	288	test	24930	36297	920
**6**	**168**	294	train	96678	139415	3599
**6**	**168**	294	test	24359	35325	893
**6**	**168**	300	train	95693	137726	3498
**6**	**168**	300	test	24153	34926	876
**6**	**168**	306	train	94117	134889	3413
**6**	**168**	306	test	23791	34251	861
**6**	**168**	312	train	91422	129977	3308
**6**	**168**	312	test	23077	33009	829
**6**	**168**	318	train	89075	126050	3222
**6**	**168**	318	test	22509	32055	805
**6**	**168**	324	train	88191	124601	3169
**6**	**168**	324	test	22280	31651	783
**6**	**168**	330	train	86611	122009	3099
**6**	**168**	330	test	21911	30987	768
**6**	**168**	336	train	83774	117018	2987
**6**	**168**	336	test	21198	29746	744
**6**	**168**	342	train	81567	113112	2901
**6**	**168**	342	test	20616	28723	722
**6**	**168**	348	train	80726	111734	2828
**6**	**168**	348	test	20412	28378	709
**6**	**168**	354	train	79350	109408	2729
**6**	**168**	354	test	20090	27822	697
**6**	**336**	0	train	682041	1773759	31465
**6**	**336**	0	test	170579	445620	7988
**6**	**336**	6	train	595230	1430806	27368
**6**	**336**	6	test	148815	359301	6959
**6**	**336**	12	train	536198	1244785	25339
**6**	**336**	12	test	133857	312142	6435
**6**	**336**	18	train	506713	1147199	23888
**6**	**336**	18	test	126598	287821	6026
**6**	**336**	24	train	470286	1026484	22583
**6**	**336**	24	test	117514	257349	5681
**6**	**336**	30	train	427620	900052	21501
**6**	**336**	30	test	107150	225732	5385
**6**	**336**	36	train	406952	846068	20459

**6**	**336**	36	test	102015	212119	5135
**6**	**336**	42	train	393103	806893	19592
**6**	**336**	42	test	98503	202406	4952
**6**	**336**	48	train	373388	749902	18808
**6**	**336**	48	test	93504	188225	4730
**6**	**336**	54	train	343796	677617	18088
**6**	**336**	54	test	86068	170102	4565
**6**	**336**	60	train	327267	644880	17414
**6**	**336**	60	test	81954	161872	4391
**6**	**336**	66	train	315030	617864	16847
**6**	**336**	66	test	78805	155133	4247
**6**	**336**	72	train	298362	577472	16188
**6**	**336**	72	test	74603	145004	4082
**6**	**336**	78	train	277859	532045	15614
**6**	**336**	78	test	69373	133613	3934
**6**	**336**	84	train	266872	510652	15027
**6**	**336**	84	test	66602	128123	3803
**6**	**336**	90	train	258273	491220	14567
**6**	**336**	90	test	64465	123099	3663
**6**	**336**	96	train	245476	460221	13999
**6**	**336**	96	test	61266	115360	3561
**6**	**336**	102	train	230507	427069	13520
**6**	**336**	102	test	57570	107056	3448
**6**	**336**	108	train	224304	414025	13105
**6**	**336**	108	test	56055	103809	3320
**6**	**336**	114	train	218128	399504	12700
**6**	**336**	114	test	54488	100198	3207
**6**	**336**	120	train	208579	376810	12291
**6**	**336**	120	test	52084	94556	3105
**6**	**336**	126	train	198380	354896	11895
**6**	**336**	126	test	49598	89216	2978
**6**	**336**	132	train	194281	346231	11584
**6**	**336**	132	test	48595	87068	2891
**6**	**336**	138	train	189601	335212	11231
**6**	**336**	138	test	47450	84279	2809
**6**	**336**	144	train	181891	317037	10884
**6**	**336**	144	test	45542	79730	2698
**6**	**336**	150	train	174289	300273	10541
**6**	**336**	150	test	43675	75626	2609
**6**	**336**	156	train	171297	294002	10237
**6**	**336**	156	test	42961	74075	2504
**6**	**336**	162	train	167556	285303	9980
**6**	**336**	162	test	42022	71882	2443
**6**	**336**	168	train	160558	269256	9663
**6**	**336**	168	test	40263	67796	2354
**6**	**336**	174	train	153786	254423	9332
**6**	**336**	174	test	38568	64092	2279
**6**	**336**	180	train	151332	249432	9086
**6**	**336**	180	test	37972	62757	2211
**6**	**336**	186	train	148059	242325	8826
**6**	**336**	186	test	37211	61047	2165
**6**	**336**	192	train	142529	230157	8558
**6**	**336**	192	test	35840	57986	2110
**6**	**336**	198	train	137562	219792	8285
**6**	**336**	198	test	34656	55452	2048
**6**	**336**	204	train	135716	216045	8089
**6**	**336**	204	test	34140	54453	1983
**6**	**336**	210	train	132996	210342	7853
**6**	**336**	210	test	33450	53001	1918
**6**	**336**	216	train	128616	201107	7589
**6**	**336**	216	test	32413	50774	1860
**6**	**336**	222	train	124902	193653	7383
**6**	**336**	222	test	31469	48941	1816
**6**	**336**	228	train	123366	190727	7174
**6**	**336**	228	test	31078	48215	1778
**6**	**336**	234	train	121165	186131	6963
**6**	**336**	234	test	30529	47120	1742
**6**	**336**	240	train	117312	178265	6750
**6**	**336**	240	test	29580	45191	1696
**6**	**336**	246	train	114069	171923	6577
**6**	**336**	246	test	28760	43594	1658
**6**	**336**	252	train	112721	169428	6427
**6**	**336**	252	test	28460	42989	1630
**6**	**336**	258	train	110669	165490	6244
**6**	**336**	258	test	27970	41993	1586

**6**	**336**	264	train	107363	158988	6081
**6**	**336**	264	test	27122	40329	1545
**6**	**336**	270	train	104592	153895	5917
**6**	**336**	270	test	26399	38991	1489
**6**	**336**	276	train	103458	151874	5763
**6**	**336**	276	test	26093	38475	1456
**6**	**336**	282	train	101827	148752	5656
**6**	**336**	282	test	25644	37637	1420
**6**	**336**	288	train	98978	143428	5522
**6**	**336**	288	test	24930	36297	1384
**6**	**336**	294	train	96678	139415	5348
**6**	**336**	294	test	24359	35325	1347
**6**	**336**	300	train	95693	137726	5195
**6**	**336**	300	test	24153	34926	1313
**6**	**336**	306	train	94117	134889	5072
**6**	**336**	306	test	23791	34251	1285
**6**	**336**	312	train	91422	129977	4916
**6**	**336**	312	test	23077	33009	1242
**6**	**336**	318	train	89075	126050	4787
**6**	**336**	318	test	22509	32055	1200
**6**	**336**	324	train	88191	124601	4683
**6**	**336**	324	test	22280	31651	1176
**6**	**336**	330	train	86611	122009	4577
**6**	**336**	330	test	21911	30987	1153
**6**	**336**	336	train	83774	117018	4430
**6**	**336**	336	test	21198	29746	1117
**6**	**336**	342	train	81567	113112	4316
**6**	**336**	342	test	20616	28723	1085
**6**	**336**	348	train	80726	111734	4220
**6**	**336**	348	test	20412	28378	1064
**6**	**336**	354	train	79350	109408	4094
**6**	**336**	354	test	20090	27822	1037
