# Supplementary material for: Development of a dynamic prediction model for unplanned ICU admission and mortality in hospitalized patients
Source: PLOS Digit Health. 2023 Jun 9;2(6):e0000116. doi: 10.1371/journal.pdig.0000116 (PMC10256150; doi:10.1371/journal.pdig.0000116)
Supplement: Attachment — Submitted filename: Response to reviewers.pdf [file pdig.0000116.s013.pdf]

Reviewer #1

Overall summary: This retrospective cohort study from Placido and colleagues aimed to develop a deep learning early warning system for clinical deterioration on the hospital wards across multiple hospitals in Denmark. The authors demonstrated high discrimination for a composite outcome of ICU admission or death.

Major Strengths

1. The authors used an extremely large cohort across multiple hospitals, which will help with generalizability and replication.

2. Isotonic recalibration is a nice approach to ensuring that the model does well across multiple performance measures (i.e., beyond just AUROC).

We thank the reviewer for pointing out these strengths of the study.

Major Limitations

1. It's not entirely clear to me where the novelty lies in this study. The Rajkomar study (ref # 14) has in some ways set the bar for hospital outcome prediction using deep learning from the EHR, and while this study purports to be more of an early warning system, designing predictions for a 14 day window is not much of an early warning. I think the study rationale/objectives should be more clearly articulated and more strongly argued.

While we fully acknowledge the relevance of the seminal Rajkomar et al. paper and the methodological advances it led to, it did not consider the outcome of unplanned ICU admission at all. The difference in the outcome definition is crucial and makes the two studies difficult to compare (mortality prediction for example could be easier if many patients are in palliative care while the opposite is the case for unplanned admission to ICU).

We note also that our study presents a dynamic risk assessment throughout the whole patient admission while Rajkomar paper limits the prediction to estimating the risk during the 24 hours before and after the admission only (for inpatient mortality). Moreover, we note that our model makes predictions for several time windows (1, 2, 7 and 14 days) while in Rajkomar et al. the prediction interval for inpatient mortality is unbounded.

Regarding the prediction window and the early warning aspect, the outcome here is defined cumulatively, i.e. the outcome can be experienced at any time between the time of assessment and 14 days. This means that the model having a prediction window of 14 days will also catch patients experiencing the outcome the next day. In addition, we also provided performance measures of models

trained using shorter prediction windows (1, 2, and 7 days) which still show good calibration (calibration curves for all models have now been included in the supplementary material).

We therefore believe that our paper has substantial novelty compared to previous work (including Rajkomar, 2018):

- A thorough interpretation of the clinical variables
- Dynamic and near-real time assessment
- Extensive performance evaluation (AUPRC curves were not included in Rajkomar for example)

Nevertheless, we agree with the reviewer that the scope and objective could be stated more clearly and therefore we have now revised the Introduction and the Discussion, but of course maintain the full acknowledgement of the Rajkomar paper.

2. The authors' methods are nice from the modeling perspective, but it's less clear how clinically operationalizable they will be. In particular, tokenizing clinical notes is fraught with uncertainty in terms of implementation and use. At what point in a real-time system would a note be "scanned" or "re-scanned"? What happens to notes written by house officers or APPs which are awaiting attending physician cosignature? What happens to notes which are revised, amended, or deleted?

While we appreciate the differences in how textual data are managed, recorded, and stored across healthcare systems and jurisdictions, we envision the real-world application to use all notes contained in the patient file that are considered valid at time of prediction:

- If a note needs co-signing, it would be sensible to not use that note until co-signed and, thus, approved as accurate,
- For amended notes, the most recent version should be preferred,
- Removed notes should therefore not be used (presuming they were removed for a reason).

This has now been expanded in the Discussion. The greatest potential hurdle is likely updating the embedding model, but regularly updating this with prospective textual data would be an obvious approach.

3. How were missing data handled in this approach?

We acknowledge that the manuscript could detail better how missing data were handled. In contrast to tabular data requiring an entry for each categorical variable using imputation, in our approach the input space is built using the available data for each patient only, so there is no missing data. However, one may encounter that some tokens are very infrequent, and the model can have problems learning from these. To account for this, we have included a parameter called 'min_freq' as part of the optimized random search: this parameter allows inclusion only if there are enough occurrences of that token, i.e. the minimum frequency for diagnosis codes is 41 tokens, which means that codes with lower frequencies were all mapped to a standard code "UNK" (unknown). We have also used padding to account for sequences with different lengths. The sequences were truncated when longer than a given padding size or expanded with the "PAD" token when shorter. To address this point (which was also raised by reviewer 2) we have expanded the text in the manuscript.

4. Lines 88-97 don't make sense to me. The authors argue that vital signs might be unreliable as EWS inputs due to limited recording, but then state that they used EHR data and clinical notes – which

are either (a) exactly those same vital signs just discounted or (b) even more sparse than ward vital signs (e.g., once-daily notes). Can the authors reconcile?

Thanks for pointing this out. We agree and as we do not have tabular vital sign data (they are embedded in the clinical notes) we found ourselves in a scenario akin to (a). We have now made this clear in the text.

5. I note that TRIPOD is mentioned in the Methods, but I do not see a relevant checklist attached in the Supplement.

The list was indeed missing, we have included the table as an additional file in the revised submission. The table could also be included in the supplementary material, but page numbering needs to be re-checked after formatting the text.

Minor comments:

1. Figure 1 is not referenced in the manuscript

We apologize for the oversight. The reference has now been included in the Introduction.

2. Line 322 seems incomplete: "performances of h is not…" (what is "h"?)

Thanks for pointing this typo out as well. We fixed it. It was originally written "Direct comparison of our model to the performances of NEWS or MEWS is not possible, …"

Reviewer #2

Development and validation of a dynamic prediction model for unplanned ICU admission and mortality in hospitalized patients

Placido et al. presented a manuscript in which they developed a dynamic prediction model with a large dataset including almost all hospitalized patients from 12 hospitals in Denmark. The authors used deep learning techniques to create the best model for the prediction of clinical deterioration. In general, the manuscript is well written and the study seems to be well performed. However, there are several concerns that need to be addressed:

Our main concern is the clinical impact of this study for the patient and/or health care provider. While reading this manuscript, the following questions arose: What is the advantage for patients/clinical care when using this model? How should the caregiver use this model in practice? What does this model add to the existing deep learning and conventional 'epidemiological' prediction models and literature? The manuscript mainly focusses on explaining their methods/the deep learning method.

We recommend to add the clinical perspective, importance and integration of the model to the manuscript.

We present a method that can run automatically based on data pulled electronically from EHRs using for example HL7/FHIR and registries. The exact real-world implementation obviously depends on the EHR system used but our paper presents proof of concept for the performance of such a model. The clinical impact of this model goes beyond its application as clinical decision support tool, as it can potentially be used for prognostic enrichment in clinical trials (enrolling patients depending on their risk of experiencing clinical deterioration) and for resource management to estimate the load of people leaving the general department (either because they are transferred to the ICU or die).

As also addressed in point 1 for reviewer 1, this model offers dynamic real-time risk assessment integrating many heterogenous data sources. Healthcare staff have a very busy daily schedule, and it is virtually impossible for them to synthesize hundreds or even thousands of data features regularly. We believe that all these aspects add novelty to the conventional epidemiological prediction models.

We have now expanded on this in the Discussion.

Our second concern relates to the data handling which is not reported. Did someone perform data cleaning on this big dataset? How were outliers handled? The authors mention in their introduction that caregivers make mistakes in manually collecting data. However, part of your data were likely to be collected or reported manually by caregivers. How much data were missing? And how did the authors handle missing data? Please add this to the manuscript.

We indeed were missing some information on how data was preprocessed. Both clinical notes and laboratory data underwent pre-processing steps, and this has now been included in the manuscript. As far as regards outliers, rare tokens were excluded using specific cut-off for each data modality (see above). Incorrect/out of range laboratory values were removed when labelled as such by the lab technician. However, we would like to stress here that we tried to develop a model able to integrate data with as little cleaning as possible, which would make the model easily implementable in real life. This may include mistakes in manually collected data that are normally difficult to correct for. Here, we crafted a performant model despite the data being almost directly pulled from EHR and with little preprocessing, making this model more operationalizable in clinical practice.

As suggested also by the first reviewer, we have expanded the section on the input data and its handling, detailing the processing done for laboratory data and clinical notes. Please also see comments to reviewer 1.

The other comments are listed below in a consecutive order:

Title:

- The authors only performed internal validation using their own dataset. Therefore, the word ‘validation’ is too broad and not appropriate in this setting. We suggest to change it to ‘internal

validation' or remove it completely. Also, the authors can provide more details on the target population. Please consider adding the country of the study population.

We understand the reviewer's point. However, the term internal validation is often used in the literature to indicate the test data, for example in cross-validation. Most readers would understand this change as if there is no holdout data used for validation at all. We made a Google search for "internal validation" AND machine learning, and the top hits reflect this practice. Although we acknowledge that the proper term would be 'internal', we would rather not change the title in this way and suggest therefore instead to remove "validation" from the title.

Abstract:

- The abstract is has been disproportionally arranged. Information about the statistical/deep learning methods used is lacking, while other parts of the abstract (introduction) are relatively long. Please rearrange the abstract and add more details on the methods and the time points used for dynamic prediction.

The abstract has been rearranged as requested.

- The final sentence of the manuscript (line 44-46) does not match the main scope and overall conclusion of the manuscript. Please rewrite this.

The conclusion of the abstract has been rearranged as requested.

- Please spell out the abbreviations used in the abstract when reported for the first time.

We have removed all abbreviations from the abstract.

Introduction:

- The introduction should start with a broader introduction of the topic. What is the importance of prediction? What are the limitations of currently used risk scores and existing deep learning models? What could the model add to clinical decision-making, i.e. what are the advantages for the patient and heath care professionals? Please explain the rationale for developing the dynamic prediction model using deep-learning and add more information on what this model could add.

We agree with the reviewer and therefore have extended and rephrased the Introduction emphasizing the importance of prediction tools in the clinic more broadly.

- The only prediction model mentioned is the EWS. However, there are many more prediction models which are often developed in a department or for a specific patient group. The authors could first write something about prediction in general, and later introduce the EWS score, as an example.

We have now provided at the beginning of the Introduction, as stated above, a broader conceptual overview of classical scores without going into the details for each of them.

- The modified early warning score (MEWS) is more often used than the EWS nowadays. Please explain why you compare to the EWS.

In the Capital region of Denmark, the score used is NEWS. To provide a wider picture of all the EWSs, we give an overview of the most important ones without comparing anyone in particular.

- Please state the research question and objectives more specifically at the end of the introduction.

We have expanded the end of the Introduction with a clearer research question.

- Please mention that this is a model development study with Internal validation. Moreover, a hypothesis is missing. Please add this.

The hypothesis is that prediction models using deeper data with many features will be useful in the clinical setting, by at the same time identifying actionable features for intervention. We have now included this in the introduction and abstract.

- Figure 1: this figure needs clarification. The 2 different examples are now difficult to distinguish. Separation of the 2 examples could be clarifying. Furthermore, the numbers/hours/days of the time points, the prediction window (particularly for ICU transfer), and outcomes used should be clarified in the figure.

The figure has been updated. Now it is mentioned exactly which different frequencies of assessment are tested and the prediction windows. In red, the assessment labelled positive because of a clinical deterioration event occurring within the prediction window, black otherwise.

Methods:

Major concerns

- In general, the deep-learning analyses seem to be performed appropriately. It would be valuable to incorporate more metrics in the supplemental material, such as the precision and recall loss. Please comment on this suggestion.

We have included in the supplementary material a full list of precision, recall and specificity for 5 prediction thresholds for the 12 models (4 prediction window, 3 assessment rates). We believe that this is sufficient to provide a wider picture of model performances.

- Please define the data used for developing the model more extensively. More information on which medical diseases, biochemical measures, and clinical notes are used in the dynamic model development is essential.

Table 1 now contains the most common comorbidities and admitting departments. Further, we have elaborated on the data sources in the appropriate section in Methods.

- There is no missing data section in the methods. Please elaborate on the number of missing data, how missings were handled, and which choices or assumptions have been made.

We agree that the missing data needed more elaboration. We have now included a section in the manuscript.

For example:

o Not all biochemical data were available every day and at the specific time points, how did the authors deal with this?

As diagnoses and clinical notes, biochemical data were extracted depending on the number of tokens available in the data. As part of the optimized random search, the optimal number of biochemical tokens to use at each assessment was 28. We agree that availability of laboratory values changes throughout the day and therefore we have included in Table 1 the statistics on the number of tokens available at three time points during the admission (we have provided similar statistics for the tokens extracted from the clinical notes, see below).

o Which clinical notes were used? Daily ones?

The clinical notes usable by the model are all the ones registered in the EHR system from the physicians (notes from nurses are not available, and we have made this clearer in the text). For clinical notes the last 299 tokens are used at each time of assessment (see supplementary Table 1). For each patient, there is usually at least one note a day. The frequency of notes recorded per day and the length of the notes are usually associated with the severity of the patient's condition. As done for laboratory tests, we have included in Table 1 statistics on the number of tokens available at three time points during the admission. This should provide enough information on the amount of data available throughout the admissions.

o How much data were missing at the specific time points? And how many patients were included at different time points?

As addressed in the section on missing values, the missing values are not present given the NLP approach used to solve the representation of input data for the prediction task. However, we have included in the Table 1 the number of tokens available at different time points during the admission. The number of patients/admissions included is constant across the different models, but at different times of assessment the number of admissions and patients changes. We have included this information in supplementary for 4 models (the frequency of assessment changes the granularity of the measurements but not the overall statistics, therefore we have listed the statistics for the model with higher frequency of assessment (6 hours) and prediction window of 1,2,7 and 14 days).

o What did the authors do with patients with a non-ICU policy? These patients are too ill, too old, or have to many comorbidities to be admitted to the ICU and could be more at risk for in-hospital mortality and theoretically ICU admission, whereas they are not allowed to be admitted to the ICU. Did the authors take this into account? Please consider a sensitivity analysis.

Thanks for raising this key point. Unfortunately, we do not have this information and we have included it under limitations.

- Table 1, there are a few comments on this table:

o Please move this table to the results section, as it describes the characteristics of the patients.

We agree, and therefore we moved the table in Results as suggested.

o Please provide more information about patient characteristics, particularly clinical data. What are the comorbidities/chronic illnesses of the patients? Which department are they from? Why are they admitted? This is a lot of information that the authors can also decide to add to the supplement.

We have extended the table including the most common comorbidities. Unfortunately, the reason for admission is not recorded in the dataset, but we have included the most common admission departments.

o Please clarify what the numbers in between the brackets mean.

We thank the reviewer for noticing that we missed this information. The distributions are in the format Median, interquartile range (25th – 75th range) - while counts are plain numbers. This has been added to the table caption.

o What does the 'Number of previous admissions' mean? How is it possible that 3 [1-7] people died in the hospital? Please clarify this.

In the example raised by the reviewer, 3 [1 – 7] are the statistics on the number of previous admissions for all the admissions having as outcome in-hospital death. We recognize that the meaning of these numbers may be ambiguous and have added a footnote in Table 1 to make their interpretation clearer.

o Has any data cleaning been performed? Did the authors check for outliers?

Data cleaning has been conducted for laboratory values and clinical notes. ICD codes are directly pulled from the Danish National Patient Registry. We agree this was not sufficiently elaborated in the manuscript and therefore, we have updated the text to describe this in greater detail. As addressed for a previous point, rare tokens were excluded using cutoff values for each data modality. Laboratory values were removed only if manually labelled by lab technicians as incorrect/out of range.

- What did the authors do with patients who died after, or in between 2 moments of, the prediction window? Are they not included? Please specify the numbers of patients that were included in each model (each frequency of assessment and prediction window) in the results.

Given an admission, the risk of developing the outcome within the prediction window is assessed at regular intervals. This means that if the outcome occurs between the time of assessment and the end of the prediction window, the patient is labelled as positive, if the outcome occurs after, the patient is labelled as negative. As the reviewer raises, for different times of assessment the number of patients and admissions decreases, as more people will experience the outcome meanwhile. For this reason, we have included a table in supplementary with these numbers.

- Did the data include patients from ALL wards, except the ICU and Emergency Department? Can the authors please specify or add a list with the wards of which patients are included? For example, did the authors also include patients from the ophthalmology and ear-nose-throat departments?

We agree this was not stated clearly in the manuscript. We have now extended Table 1 with the most common admission departments which comprise emergency medicine departments (generally receiving acutely ill patients). The only departments that have been removed are the ICU departments and acute admissions to the emergency department (inpatients admission to the emergency department were kept).

- Please add information on who assessed or reported the outcome measures? Were those researchers blinded?

Given the retrospective nature of the study design (notwithstanding the prospective nature of data collection), there was no blinding. We use real-world routine data and did base outcome variables on structured data on ICU transfer (specific procedure codes) and death (from relevant register).

Minor concerns:

- Line 113: please specify the definition of 'direct ICU admissions'. Are those patients admitted within hours? Or directly?

We have used a window of 24 hours to consider the ICU admission a transfer from another department. This means that ICU admissions not preceded by any admission within 24 hours were considered direct admissions and therefore excluded. We have rephrased this to make it clearer.

- Line 120: this is unclear. It seems that those patients have already been discharged and are then admitted again. Please reformulate or clarify this.

This somehow relates to the point above. We have used a 24-hour window to capture transfers across departments. If an unplanned ICU admission occurs within 24 hours from any previous discharge, then it is considered part of the same admission, otherwise it is considered direct admission to ICU and therefore discarded. We have reformulated this in the manuscript.

- Line 121: please adjust the title of this paragraph. A suggestion could be 'model development'.

We agree that a better name for this paragraph is indeed 'model development'. We have modified it.

- Figure 2: please enlarge this figure, the figure is difficult to read.

We agree it is very difficult to read. Therefore, we have expanded and included a high-quality figure in the revised submission.

- Line 189-194: maybe the authors can add a figure to the supplements to visualize this process.

We agree a figure would help the understanding of the process and dataset split, and we therefore have included one in the supplementary material (Figure S7).

Results:

- Please add data on how many patients were included per prediction window and per time point? This is interesting because a lot of patients are in the hospital for less than 14 days. What happened if

a patient died or was discharged outside the prediction model? This is also stated in the TRIPOD guidelines, 14a: 'Specify the number of participants and outcome events in each analysis.'

We agree it is important to make that clear. If the admission is shorter than 14 days and within the admission the patient does not die/admitted to the ICU, the patient is labelled as not experiencing the outcome (clinical deterioration). As addressed in a previous point, for different time of assessment the number of patients and admissions decreases, as more people will experience the outcome in the meanwhile. We have extended the Supplementary with a table, as mentioned above.

- Calibration is a very important component of the predictive performance of a prediction model. Figure S5, the calibration plot of the best performing model, should be replaced to the main paper. In addition, please visualize all other calibration plots in the supplement. Furthermore, please add more textual information about the calibration of the models and their interpretation, as this is now underreported.

We agree calibration is an extremely important aspect and therefore we have moved it to the main text.

- Please explain more why the 6-hour/14-day model is the best model. This is not clear now as it has not the highest AUROC or best calibration. Only the AUPRC, as a measure of discrimination, is highest.

We briefly mentioned this in the Training and evaluation section, by saying that AUPRC was used to select the best model. Nevertheless, we agree this decision was not mentioned in the Results section and now has been included. There is no ground rule on which measure is the best, but given the imbalance of the dataset, in this case we believe that AUPRC is the more robust and effective measure of discrimination.

- Figure 4DEF: these figures are very interesting and important, and should be highlighted more. Please adjust these, since there are unreadable.

We are aware of the poor quality of the initial figures. Unfortunately, embedding the figures directly in the .docx document compressed them to the point of rendering them near-illegible. We have changed the settings so that the original quality is preserved, and in addition, we have now included original vectorized figures in the submission such that the problem is eliminated.

Discussion:

Major concerns

- In the discussion, the authors should focus more on the clinical interpretation. Why is this model better than already existing/developed models and how can we apply the model in daily patient care? How do the authors envision this?

Thank you for stressing this important point. Overall, we believe that the clinical interpretation of the model is covered by the section "Interpretation" (see also next comment). Please also note that the main aspect of the model is that it can handle numerous features and, in this way, many different subgroups of patients at risk of experiencing the outcomes. We anticipate that models like this quite easily can be implemented in EHR systems; we work in this domain and know about the real time barriers and the need for regulatory approvals - we now mention this implementation aspect briefly. We

have already addressed this concern in a previous point; we have also expanded the Discussion with a clearer overview of the advantages of this model and perspectives on clinical implementation.

- The paragraph 'interpretation' should be added to the limitations paragraph, as the content of this paragraph contains limitations instead of interpretations.

We respectfully disagree with this suggestion. The interpretation section deals with the fundamental nature of explainability and not with limitations of the general method. Obtaining non-causal explanations is a direct consequence of the study design (being a prediction study, not etiological) and not a limitation of the method as such. Indeed, the primary benefit of xAI explanations is to enable sanity checks to ascertain that the model has picked up meaningful signals. We believe this section does exactly this, and does it well, drawing also on clinical insights and counterintuitive examples. Thus, we believe this section works well on its own and note that reviewer #1 has not raised this point.

- Limitations:

o The authors describe that there are many errors in the manual collection of data, but there are also errors in data that has been pulled directly from the system. How did they deal with this?

Indeed. This is a fundamental shortcoming of real-world data. As addressed in a previous point, manual curation was carried out for clinical notes and laboratory values. This cleaning was done at the cohort level, with the assumption that errors at the patient level would be handled implicitly by the model, thanks to the size of the dataset used for development and validation. Considering the promising performance, a natural next step would be testing the model in a randomized setting to see if these in-silico results translate into real "in-clinico" benefits to the patients.

o Vital signs have been left out, but are very important for mapping a patient, assessing clinical deterioration/improvement and estimating ICU requirement. In addition, they are likely to be measured at all departments. Regardless of whether vital signs are included in the model, it is important to still measure vital signs as clinical parameter. Please add this to the limitations.

Indeed; the manuscript now discusses this under Limitations.

o Add a section regarding missing data.

Although we briefly touched on this, we agree that the manuscript would benefit from a more detailed description (as also mentioned by Reviewer #1) and have, consequently, expanded in the Methods section.

- Strengths: this model differs from other models, because it does not select a specific patient group because with a certain disease or at a specific department. We assume that all patients admitted to all departments are included. Existing models often aim to predict at a certain group of patients or a certain department. This is a strength of the model that could be emphasized.

Thanks for highlighting this strength, which we have added to the manuscript.

- Please discuss the potential clinical use of the model and implications for future research in a new paragraph. Consider external validation.

We agree and we have expanded this in the conclusion. We have also stressed the future need for external validation, for example in a trial setting.

- The conclusion should apply more directly to the study, presenting the best model, results, and implication of this study. Please incorporate this.

We agree and we have expanded this following this advice.

Minor concerns:

- Line 316: number 0.28 is incorrectly rounded, please change it to 0.29

Thanks. This has been corrected.

- Line 322: there is one word or there are a few words missing in this sentence. What does h mean? Please rewrite.

As pointed out also by the first reviewer, there was indeed a typo. It has been corrected to read “Direct comparison of our model to the performances of NEWS or MEWS is not possible,… ”
